# Universality of the electrical transport in granular metals

**DOI:** 10.1038/srep29676

**Published:** 2016-07-14

**Authors:** Hicham Bakkali, Manuel Dominguez, Xavier Batlle, Amílcar Labarta

**Affiliations:** 1 Departamento de Fisica de la Materia Condensada and Instituto de Microscopía Electrónica y Materiales, Universidad de Cadiz-11510 Puerto Real, Cadiz, Spain; 2 Departament de Física de la Matèria Condensada and Institut de Nanociencia i Nanotecnologia (IN2UB), Universitat de Barcelona (UB)-Avinguda Diagonal 647, 08028 Barcelona, Catalonia, Spain

## Abstract

The universality of the ac electrical transport in granular metals has been scarcely studied and the actual mechanisms involved in the scaling laws are not well understood. Previous works have reported on the scaling of capacitance and dielectric loss at different temperatures in Co-ZrO_2_ granular metals. However, the characteristic frequency used to scale the conductivity spectra has not been discussed, yet. This report provides unambiguous evidence of the universal relaxation behavior of Pd-ZrO_2_ granular thin films over wide frequency (11 Hz–2 MHz) and temperature ranges (40–180 K) by means of Impedance Spectroscopy. The frequency dependence of the imaginary parts of both the impedance *Z*″ and electrical modulus *M*″ exhibit respective peaks at frequencies *ω*_*max*_ that follow a thermal activation law, ***ω***_***max***_** ∝ exp(*****T***^**1/2**^). Moreover, the real part of electrical conductivity *σ*′ follows the Jonscher’s universal power law, while the onset of the conductivity dispersion also corresponds to *ω*_*max*_. Interestingly enough, *ω*_*max*_ can be used as the scaling parameter for *Z*″, *M*″ and *σ*′, such that the corresponding spectra collapse onto single master curves. All in all, these facts show that the Time-Temperature Superposition Principle holds for the ac conductance of granular metals, in which both electron tunneling and capacitive paths among particles compete, exhibiting a well-characterized universal behavior.

Disordered dielectric materials, consisting of random distributions of conducting and insulating phases, are present in a broad variety of systems such as ceramic composites, polymers, semiconductors or thin films of nanostructured granular metals. The latter have been extensively studied due to their relevant physicochemical properties^1^,^2^,^3^,^4^,^5^,^6^,^7^, promising optical^8^,^9^ and several sensing applications^10^,^11^,^12^. Most of those materials display a similar conductivity-frequency dependence as proposed by Jonscher and known as the universal power law (UPL)^13^,


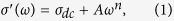



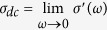
 being the *dc* conductivity of the material, *A*, a pre-exponential constant and *n*, a fractional exponent which is typically 0 < *n* < 1. Both *A* and *n* are temperature dependent. The *ac* conductivity of these materials is also found to follow a universal dynamic process, since temperature only changes the number density of charge carriers in ionic conductors^14^,^15^ or the electron hopping probabilities in granular metals^16^,^17^. Jonscher’s UPL model successfully describes most of disordered dielectric materials when the *ac* conductivity and permittivity show a dispersive region beyond a critical frequency^18^,^19^. Several authors^19^,^20^,^21^,^22^,^23^ have applied this model to examine the electrical response of random resistor-capacitor networks, which easily model a heterogeneous microstructure consisting of randomly distributed conducting and insulating regions. Moreover, it has been shown in the case of glasses, amorphous semiconductors and polymers that the conductivity spectra collapse into a single master curve, suggesting the validity of the so-called Time-Temperature Superposition Principle (TTSP), which can be expressed by the following scaling law^13^,^15^,^24^,^25^,^26^,^27^:


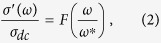


where *F* is a temperature-independent scaling function and ω* is the characteristic frequency corresponding to the onset of the conductivity dispersion, which in turn is the scaling parameter. Hopping conduction is proposed by many studies in order to account for the characteristic frequency ω* acting as the scaling parameter for all conductivity spectra in the measured temperature range^28^,^29^. In such works, the onset of the conductivity dispersion is attributed to the relaxation processes related to both grain and grain boundary contributions^30^. Nevertheless, in the case of granular insulating thin films in the dielectric regime, the *ac* transport is originated from electron tunneling and capacitive paths among particles in the sample, rather than from ion hopping. Furthermore, the relaxation processes in granular metals are related to competing electron tunneling and capacitive paths among nanoparticles in the material. Previously, it was reported by some of us that capacitance and dielectric loss show scaling behavior in granular metals^31^. Consequently, the characteristic frequency corresponding to the onset of the conductivity dispersion has been attributed to the frequency at which the magnitudes of the admittances of both the tunneling and capacitive paths become similar^4^,^7^,^31^,^32^,^33^. In this report, we determine independently the critical frequency ω* by analyzing the dielectric impedance *Z*″, electrical modulus *M*″, and the ac conductivity spectra *σ*′, respectively, of Pd-ZrO_2_ granular metal up to room temperature and over a wide frequency range (11 Hz–2 MHz) and we show the scaling behavior of all three spectra at low temperature by applying the TTSP and Jonscher’s UPL models.

## Experimental

Pd-ZrO_2_ samples were evaporated on SiO_2_ substrates by co-sputtering of Pd and ZrO_2_ stabilized with 7% volume yttrium oxide (Y_2_O_3_) targets, using magnetron sputtering (MS). The distance between the target and sample was fixed at 10.7 cm, argon pressure was 2.3 × 10^−5^ bar, sample exposure time was 30 second, film thickness was 10 nm and the deposition power was 30 Watts for Pd and 255 Watts for ZrO_2_. After deposition of the Pd-ZrO_2_ layer, four parallel Au strips were thermally evaporated on the sample surface to provide the metallic contacts for four-point electrical measurements. These strips were approximately 5 mm long, 1 mm wide, and were separated by a distance of 0.7 mm. The films were initially characterized with a JEOL 2010 high resolution transmission electron microscope (HRTEM) for structural analysis as shown in ref. 32. A bimodal size distribution of Pd particles is observed, with one peak centered at smaller sizes (~2 nm) corresponding to most of the Pd nanoparticles and the other (less clear, shown as a shoulder) at larger sizes (>5 nm) corresponding to fewer Pd nanoparticles (less than about 10% of the overall number of particles^32^). The average distance among the smaller particles is of about one nanometer, which is excellent for tunneling conductance^34^,^35^. Elemental analysis was performed by Energy Dispersive X-ray microanalysis (EDX), leading to a metallic volume fraction of *x* = 0.28. The contribution to the capacitive conductance is considered to be dominated by the larger particles, which have much smaller impedance than that of the smaller particles^32^. The modulus of complex impedance (|*Z(ω*)|) and the phase angle were measured in the frequency range of 11 Hz to 2 MHz and in the temperature range of 40–280 K, using a precision LCR Meter (QuadTech 7600 Plus), a KEITHLEY 6221 AC current source and a KEITHLEY 2182A Nano-Voltmeter in a continuous-flow He cryostat.

## Results and Discussion

**Figure 1:**
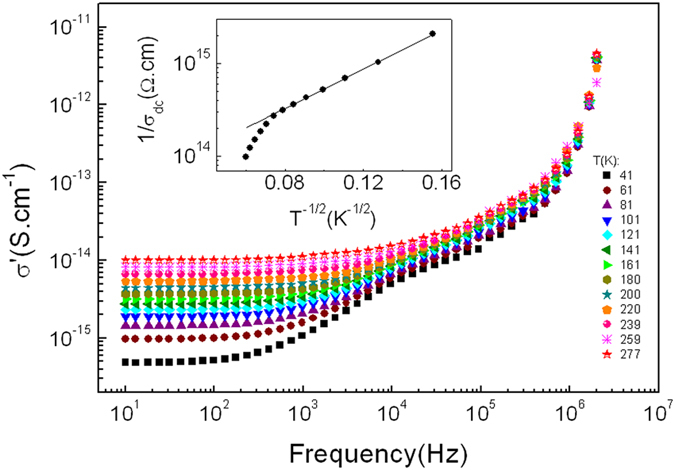
Plot of real part of the conductivity *σ*′ as a function of frequency in a log-log scale in the temperature range of 40–280 K. The inset shows the *dc* resistivity *ρ*_*dc*_ = 1/*σ*_*dc*_ (obtained from extrapolation of *σ*′ to zero frequency) versus *T*^−1/2^ in a semi-log scale. The solid lines are linear fits.

The real part of the conductivity *σ*′ for Pd-ZrO_2_ is calculated using:


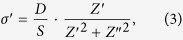


where *Z*′, *Z*″ are, respectively, the real and imaginary parts of impedance, 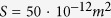 is the electrode area, and 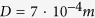 is the inter-electrode separation. *σ*′ is plotted versus frequency at different temperatures using a log-log scale in Fig. 1 and exhibits two different regions as function of temperature: (i) a dc plateau region and (ii) a frequency-dependent region, which is due to the competition between tunneling and capacitive paths among Pd particles embedded within the ZrO_2_ amorphous matrix^32^. This observation is in accordance with Equation (1) proposed by Jonscher. Here, it should be noted that, at very high frequencies (*f* ≥ 100 *kHz*), *σ*′(*ω*) increases much faster than expected. This anomalous behavior is not caused by any intrinsic phenomenon in the granular system but it arises from the significant contribution at such high frequencies of the measuring leads and electrodes, leading to high electrical losses^36^,^37^. Besides, it has been shown in previous works^4^,^7^,^31^,^32^,^33^ that, in the case of Pd-ZrO_2_ granular metals, the onset of the conductivity dispersion corresponds to the frequency at which the magnitudes of the admittances associated with resistive (tunneling) paths between the smallest Pd particles (size ~2 nm), which make up the dc tunneling backbone of the sample, and those associated with the capacitive paths among larger Pd particles (size >5 nm), become similar. This dispersive regime is shifted towards high frequency until it disappears when temperature approaches room temperature (~280 K). The dc resistivity *ρ*_*dc*_ = 1/*σ*_*dc*_ has been obtained from the extrapolation of the plateau region to zero frequency and is plotted versus *T*^−1/2^ in a semi-log scale in the inset to Fig. 1. At low temperatures, 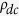 is well described by the inelastic co-tunneling mechanism^17^,^38^ for granular metals following thermal activation 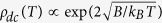, which was previously pointed out by Abeles^39^. The activation energy *B* is directly related to the Coulomb charging energy of the particles *E*_*C*_, where *B* = *E*_*C*_*dχ*, 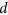 being the interparticle distance associated with the smallest Pd particles and 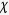 the tunneling barrier energy in units of wave vector. From the linear fit (inset to Fig. 1), we obtain an experimental value of 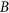 = 12.6 meV. However, at T > 180 K, the *dc* resistivity deviates from the linear relationship, due to the fact that thermal energy *k*_*B*_*T* overcomes the charging energy of the particles *E*_*C*_^40^,^41^.Therefore, *ρ*_*dc*_ can no longer be accounted for by the former simple exponential law in the temperature range 200–280 K. Thus, we have focused our study on the temperature range 40–180 K, where the conditions for thermal activated tunneling conduction are fulfilled.

**Figure 2:**
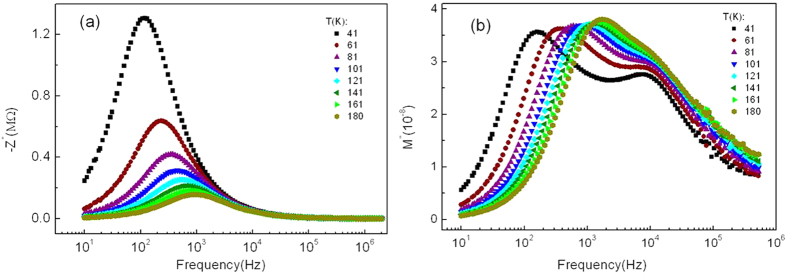
(**a**) Imaginary part of impedance *Z*″ as a function of frequency in a semi-log scale, (**b**) Imaginary part of the electrical modulus *M*′ as a function of frequency in a semi-log scale. Two peaks are observed in the spectra, at low and high frequency, respectively.

The electrical impedance has been analyzed by plotting the imaginary part of impedance *Z*″ versus frequency in a semi-log scale at different temperatures (Fig. 2(a)). This plot provides information on the dielectric processes taking place in the material. One peak at *ω*_max_ is observed in *Z*″ versus frequency which is shifted to higher frequency with increasing temperature, indicating the existence of relaxation processes in the system, while its broadening on increasing temperature suggests that those relaxation processes are temperature-dependent^13^.

**Figure 3:**
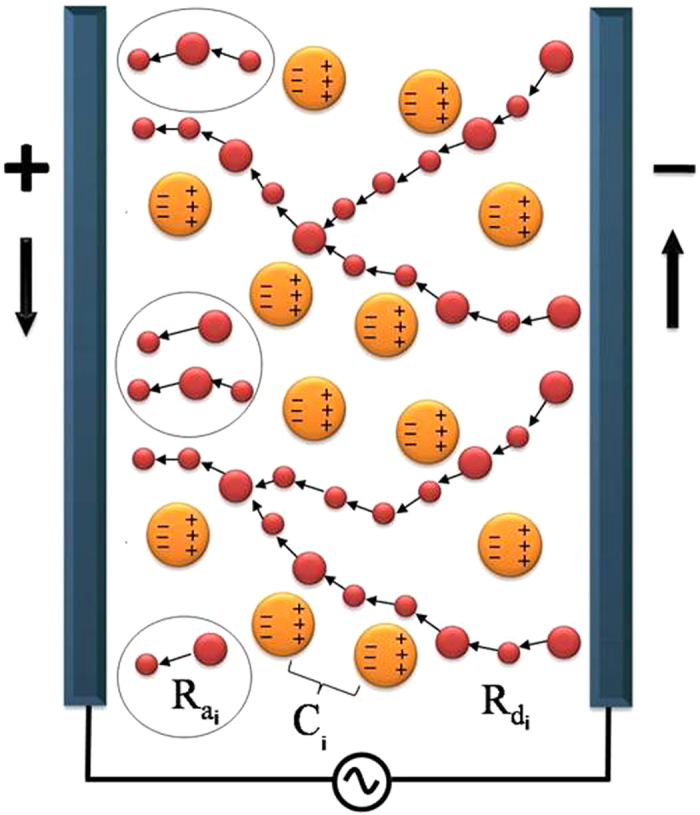
Simplistic sketch of the ac electrical conductance model in Pd-ZrO_2_ granular thin film in the dielectric regime. At low frequency, most of the smallest Pd particles are electrically connected by the dc tunneling backbone 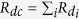, whereas, at ~1 kHz, an additional contribution of assisted tunneling resistive paths 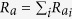 among smaller particles, initially isolated at low frequencies, improves the electrical conductance. The polarized bigger particles only contribute to the capacitive conductance C_i_ due to the large separation from each other.

The electrical response of Pd-ZrO_2_ has also been analyzed using the complex electrical modulus formalism. This method is useful for elucidating the relaxation mechanisms in a material having different magnitudes of resistance and/or capacitance^42^,^43^. The imaginary part of the electrical modulus is calculated by using the relationship:


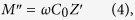


where 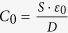, and *ε*_0_ is the permittivity in free space. Figure 2(b) shows the semi-log plot of *M*″ versus frequency at different temperatures, where two peaks in *M*″ are observed, indicating the occurrence of two relaxation processes. Those two relaxation mechanisms can be explained in terms of the competition between parallel tunneling and capacitive paths among the metallic particles, as a consequence of the bimodal size distribution of the Pd particles, explained in more detail elsewhere^32^. Considering this bimodal size distribution with a larger population of the smaller particles, Fig. 3 shows a simplistic sketch model of the ac electrical conductance in Pd-ZrO2 granular thin film in the dielectric regime. At low frequency, most of the smallest Pd particles are electrically connected through effective resistances due to thermally-activated tunneling. They form the so-called dc tunneling backbone of the sample, whose equivalent resistance is given by 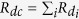, 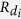 being the tunneling resistance among two small particles. However, there is a certain amount of these smaller particles that are isolated from the dc tunneling backbone at these low frequencies. On the other hand, the set of the largest particles are only connected by capacitive paths *C*_*i*_, being 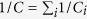. At intermediate frequencies (~1 kHz), the larger particles are short-circuited and these capacitive shortcuts provide paths to connect the previously isolated smaller particles to the electrical backbone. Thus, an additional contribution to resistivity 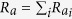 arises from this so-called assisted-tunneling process. In this case, 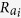 is the tunneling resistance among two small particles that become connected to the backbone at this frequency. Here, the low frequency peak *ω*_1*max*_ in Fig. 2(b) corresponds to the frequency at which the magnitudes of the admittances of the dc tunneling backbone resistance 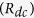 and capacitive paths (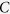) become similar, i.e., 
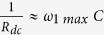
. However, the high frequency peak *ω*_2max_ in Fig. 2(b) corresponds to the frequency at which the magnitudes of the admittances associated with assisted tunneling resistance paths (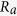), and the capacitive paths (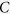) become similar, i.e., 
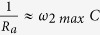
. In this case, it is worth noting that *R*_*a*_ ≪ *R*_*dc*_ since the number of the smaller particles involved in the assisted tunneling process at intermediate frequencies is much smaller than the number of those involved in the dc tunneling backbone. Therefore, the *ac* response of the sample is assumed to be dominated by the dc tunneling backbone resistance and, as a consequence, the high frequency peaks are not observed in the impedance spectra (see Fig. 2 (a)).

**Figure 4:**
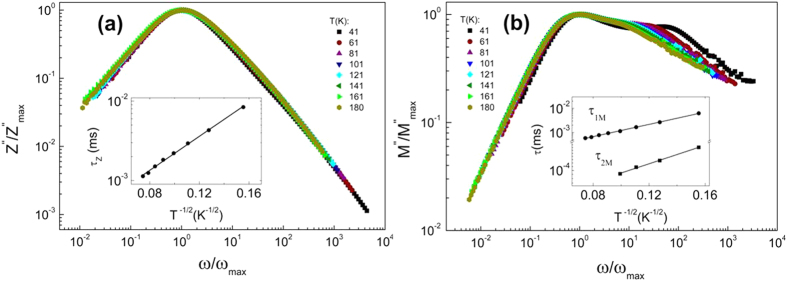
(**a**) Scaling plot 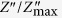 versus *ω*/*ω*_max_ in a log-log scale. The inset shows the variation of the frequency of the peak in *Z*″ as a function of *T*^−1/2^ in a semi-log scale and (**b**) Scaling plot 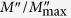 versus *ω*/*ω*_max_ in a log-log scale. The inset shows *τ*_1*M*_ and *τ*_2*M*_ as a function of *T*^1/2^ in a semi-log scale. *τ*_1*M*_ is the low frequency and *τ*_2*M*_ is the high frequency relaxation times, respectively, obtained from the peaks in the main figure. The solid lines are linear fits.

The scaling behavior of the electrical impedance was studied by plotting *Z*″/*Z*″_max_ versus *ω*/*ω*_max_ in a log-log scale (see Fig. 4(a)). All *Z*″ curves collapse onto a single master curve following a power law behavior with frequency, both above and below the frequency of the peak, *ω*_max_. This suggests the presence of an absorption process^13^,^18^ for which all relaxation times *τ*_*Z*_ occurring at different temperatures exhibit the same activation energy *U*_*Z*_, indicating that the dynamic process is temperature-independent^44^. The inset to Fig. 4(a) shows the variation of relaxation time *τ*_*Z*_ as a function of temperature, calculated using the relationship 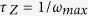. Those relaxation times exhibit a characteristic temperature dependence expressed as 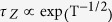. Moreover, we showed in a previous work^32^ that the relaxation process in Pd-ZrO_2_ granular metals occurs when the condition 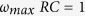 is fulfilled, i.e., the magnitudes of the admittances of the tunneling and capacitive paths among particles become similar. Therefore, it is reasonable to expect that 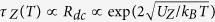, since the effective capacitance 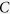 through the set of the bigger particles is temperature-independent^31^,^32^. To confirm this, we obtained the activation energy *U*_*Z*_ value from the slope of the linear fit in the semi-log plot of the inset to Fig. 4(a), yielding an experimental value of *U*_*Z*_ = 13 meV. This value is in agreement with 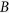 = 12.6 meV obtained from *ρ*_*dc*_.

Furthermore, the scaling behavior of the sample at low frequency is analyzed by plotting the normalized value of *M*″, i.e., *M*″/*M*″_max_, versus 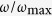 in a log-log scale (see Fig. 4(b)), where *ω*_max_ corresponds to the low frequency peak *ω*_1max_ and 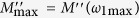. The normalized spectra around the high frequency regime is not shown since the relaxation frequency of assisted tunneling resistance paths *R*_*a*_ is assumed to be negligible with respect to that of the dc tunneling backbone^32^. Similar scaling behavior to that previously observed in the peak position of *Z*″ is shown for *M*″. The comparison of the data for the impedance and electrical modulus (see Table 1) shows that the peaks observed at low frequency in *M*″ nearly match the *Z*″ peaks, suggesting that both peaks are due to the same relaxation process which dominates the conductance. The slight shift observed in the *M*″ peaks toward higher frequencies with respect to the *Z*″ peaks is a mathematical result of applying Equation (4) to impedance data in order to calculate the electrical modulus^45^ (see Table 1). The collapse of all the curves at different temperatures (either *Z*″ or *M*″) onto a single master curve indicates that TTSP is fulfilled, and that 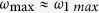 is the proper choice as scaling parameter. Consequently, the ac electrical transport properties of granular thin films in the dielectric regime show a well-characterized universal behavior.

Both low frequency 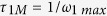 and high frequency 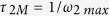 relaxation times are plotted as a function of temperature in the inset to Fig. 4(b) showing that they are well described by the Abeles model since they follow the characteristic 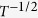 dependence. It is thus expected that 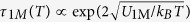 and 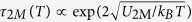, where 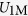 and 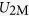 are the activation energies corresponding to 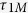 and 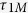, respectively. From these linear fits we may extract the values of 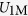 = 12.5 meV and 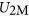 = 0.95 meV. The fact that the values of 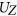 and 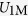 obtained from *Z*″ and *M*″ spectra are very much the same confirms that the relaxation frequency *ω*_*max*_ associated with the dc tunneling backbone completely dominates the ac response of the sample, following 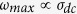.

**Figure 5:**
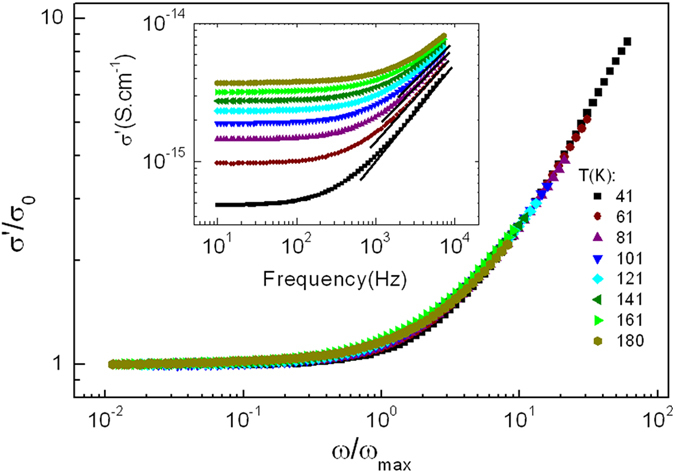
Scaling plot of *σ*′/*σ*_*dc*_ versus *ω*/*ω*_max_ in a log-log scale in the temperature range 40–180 K. The inset shows a log-log scale of *σ*′ versus frequency in the frequency range of 11 Hz–10 kHz. Solid lines indicate the fit of the data to a power law with a fractional exponent *n*.

Finally, Fig. 5 shows 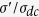 versus 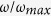 in a log-log plot, demonstrating that conductivity also scales with *ω*_*max*_. Experimental data are plotted up to 10 kHz where the Jonscher’s UPL is fulfilled. The power law of *ω* is observed above the dc plateau region for all temperatures. According to Equation (1), the *ac* contribution to the conductivity can be written as 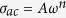, where the exponent *n* gives the curvature of the dispersive region. The *n* values obtained from the linear fit of the log-log plot of *σ*′ versus *f* (solid lines in the inset to Fig. 5) at different temperatures are shown in Table 1. These values are observed to decrease with increasing temperature. This observation agrees with the case of heterogeneous materials consisting of random distributions of conducting (resistor) and insulating (capacitor) phases, where the parameter *n* in the power law is directly related to the fraction of capacitive paths involved in the *ac* conduction mechanism^19^,^20^,^21^,^22^,^23^. In our case, the *ac* response of the sample arises from the competition between the *dc* tunneling backbone resistance *R*_*dc*_ between smaller particles, and the capacitive reactance *X*_*C*_ among bigger particles, 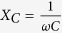, the latter being temperature-independent. In contrast, the resistive paths follow thermal activation, 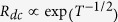 and, therefore, increasing temperature, *R*_*dc*_ decreases. As a consequence, the resistive paths are more favored than capacitive ones and hence *n* decreases with temperature.

The fact that the conductivity curves in Fig. 5 collapse onto a single master curve with an exponent *n* of 0.71 indicates that TTSP is fulfilled and suggests that the distribution of relaxation times is temperature independent^44^. Furthermore, the conductivity formalism indicates that *ω*_*max*_ may be regarded as the critical scaling parameter. This confirms our assumption concerning temperature-independent *C*, since the characteristic frequency that corresponds to the onset of the conductivity dispersion is found to be the same as the relaxation frequency of the tunneling resistance, i.e., 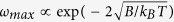. Finally, the power law exponent *n* extracted from the dispersive region in Fig. 5 (*n* = 0.71) is in good agreement with the empirical value of the universal law of Jonscher which should be smaller than one^19^,^22^,^46^,^47^,^48^.

## Conclusions

The scaling behavior of the ac electrical response of the Pd-ZrO_2_ granular metal in the dielectric regime has been studied in the temperature range 40–180 K. We find that the imaginary part of both the impedance *Z*″ and the electrical modulus *M*″, and the real part of the conductivity *σ*′ spectra collapse onto single master curves using a characteristic frequency *ω*_max_ as a scaling parameter. This frequency is associated with the main relaxation processes in the system, which manifests itself through the appearance of low frequency peaks in *Z*″ and *M*″, and a dispersive regime in *σ*′, and it is attributed to competing tunneling and capacitive paths among Pd particles in the amorphous ZrO_2_ matrix. This scaling behavior confirms the validity of the so-called Time Temperature Superposition Principle (TTSP) for granular metals. Furthermore, the frequency-dependent conductivity *σ*′ is found to obey the universal power law proposed by Jonscher, with a universal exponent value of 0.71, which agrees with the typical exponents, smaller than one. In summary, the ac electrical transport of Pd-ZrO_2_ granular metal in the dielectric regime is shown to follow a universal behavior when thermally activated tunneling dominates the conduction mechanism, i.e., 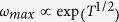.

## Additional Information

**How to cite this article**: Bakkali, H. *et al*. Universality of the electrical transport in granular metals. *Sci. Rep.* **6**, 29676; doi: 10.1038/srep29676 (2016).

## Figures and Tables

**Table 1:** Experimental values of *ω*_max_ extracted from *Z*″, and *ω*_1max_ and *ω*_2max_ extracted from *M*′, and power law exponent *n* extracted from *σ*′ in the dispersive region.

*T*(*K*)	*ω*_max_(*rad*/*s*)	*ω*_1max_(*rad*/*s*)	*ω*_2max_(*rad*/*s*)	*n*
41	748	999	45113	0.71
61	1458	2350	54582	0.65
81	2136	3782	60042	0.59
101	2846	5542	66049	0.55
121	3443	6704	–	–
141	4166	8111	–	–
161	5039	9814	–	–
180	5542	10800	–	–
